# Study on the Mass Spectrometry Cleavage Pattern of Quinolone Antibiotics

**DOI:** 10.1002/open.202400061

**Published:** 2024-06-17

**Authors:** Susu Fan, Hui Lu, Chunjian Li, Meng Cai, Jian Shi

**Affiliations:** ^1^ Nantong University Seyuan road No.9 Nantong Jiangsu China E-mail address; ^2^ Qidong Secondary School of Jiangsu Province Qixiu road No.536, Qidong Nantong Jiangsu China

**Keywords:** quinolone, antibiotics, mass spectrometry, cleavage pattern

## Abstract

Rationale: Quinolone antibiotics are extensively used clinically for human treatment and in agriculture. However, improper and excessive use can lead to the persistence of quinolone residues in animal tissues, potentially accumulating in the human body and posing health risks. Investigating the correlation between mass spectrometry cleavage patterns and molecular structural features enhances the analytical framework for detecting trace or unknown impurities in quinolones. Methods: To collect data, we employed triple quadrupole linear ion trap mass spectrometry in electrospray positive ion mode. Primary mass spectrometry scanning was utilized to confirm parent ions, while secondary mass spectrometry scanning enabled the observation of fragment ions. The cleavage characteristics and pathways of the compounds were inferred from accurate mass‐to‐charge ratios obtained from both primary and secondary mass spectrometry. Results: Under soft ionization conditions, the compounds generally exhibited characteristic fragment ions of [M+H−H_2_O]^+^, [M+H−CO]^+^, and [M+H−H_2_O−CO]^+^. Additionally, subtle variations were observed in each compound due to differences in modifying groups. For instance, upon deacidification, the piperazine ring structure underwent breakage and rearrangement, yielding fragment ion peaks devoid of neutral molecules such as C_2_H_5_N, C_3_H_7_N, or C_4_H_8_N. Notably, compounds featuring a cyclopropyl substituent group at the N‐1 position typically exhibited characteristic fragments resulting from the loss of the cyclopropyl radical (⋅C_3_H_5_). Moreover, substituents at the N‐1 and C‐8 positions, when linked to form a six‐membered carbocyclic ring, were prone to cleavage, releasing the neutral C_3_H_6_ molecule. Conclusion: Quinolone antibiotics share structural similarities in their parent nuclei, leading to partially similar cleavage pathways. Nevertheless, distinct cleavage patterns emerge due to variations in functional groups. According to the difference of mass spectrometry cleavage patterns, it can provide an identification basis for the measured detection of antibiotics.

## Graphical abstract

Quinolone antibiotics share similar parent nuclei and partially similar cleavage pathways, their distinctive functional groups induce varied cleavage patterns. These characteristic fragment ions provide an essential basis for the structural analysis of quinolone antibiotics with identical mother nucleus structures, facilitating the rapid screening and structural identification of novel quinolone antibiotics using mass spectrometry techniques.

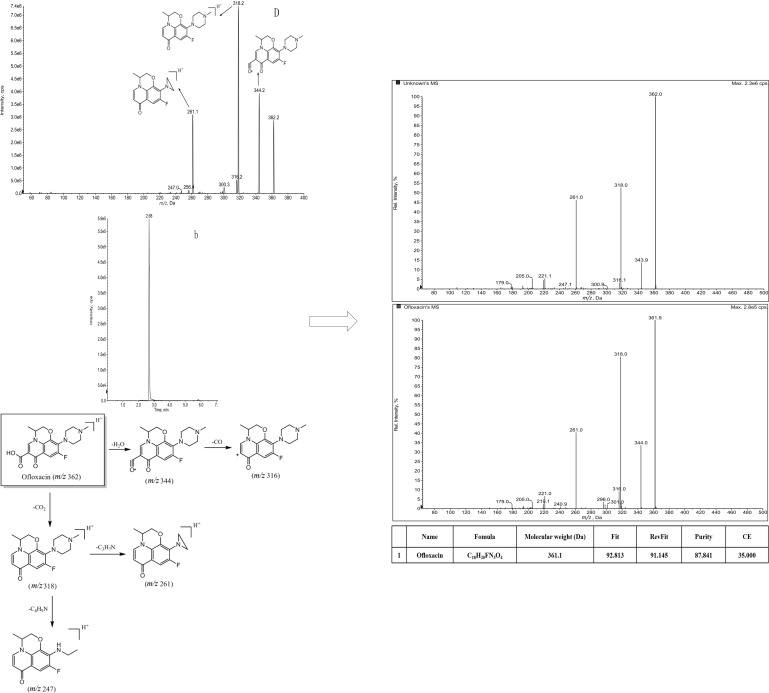

## 1 Introduction

Quinolone antibiotics constitute a class of synthetic antimicrobial drugs characterized by a 4‐quinolone as their core nucleus and nitrogen (hetero)bipyramidal structure as their basic skeleton. These antibiotics and other antimicrobial drugs exert their effects primarily by targeting the bacterial deoxyribonucleic acid (DNA). Notably, quinolones boast a broad antibacterial spectrum,^[1]^ robust antibacterial efficacy, minimal cross‐resistance with other antibacterial agents, and limited toxic side effects.[^2^, ^3^, ^4^] Consequently, they find widespread use in disease management within the aquaculture sector.[^5^, ^6^, ^7^] Nalidixic acid represents the first generation of quinolones,[^8^, ^9^, ^10^, ^11^] while subsequent generations have seen expansions in the antimicrobial spectrum and modifications aimed at enhancing efficacy and reducing toxicity. This evolution includes the introduction of fluorine atoms and the incorporating of the piperazine ring in the second and third generations, respectively.^[12]^

Structural investigations into quinolone antibiotics, extensively documented in the literature, reveal that alkyl group modifications at the N‐1 position of the 4‐quinolone parent nucleus can augment antimicrobial properties and bolster activity against Chlamydia and Mycoplasma. Moreover, the carboxylic group at the C‐3 position and the carbonyl group at the C‐4 position are deemed essential for their antimicrobial efficacy.^[13]^ Substitution of the amine group at the C‐5 position enhances overall antimicrobial activity,^[14]^ while fluorine substitution at the C‐6 position significantly impacts the antimicrobial properties and bactericidal effect of the drug.^[15]^ The presence of the piperazine ring at the C‐7 position enhances intestinal absorption,[^16^, ^17^] improves cell penetration, and prolongs drug half‐life while introducing halogen atoms at the C‐8 position enhances absorption properties and the antioxidant capacity of orally administered drugs.

Quinolone antibiotics, derived from the 4‐quinolone parent nucleus, feature various functional groups at distinct sites. Even compounds with similar structures exhibit different cleavage patterns due to the positions of substituent groups, resulting in complex cleavage patterns during collision‐induced dissociation. This paper investigates the cleavage patterns of eleven quinolone antibiotics, including nalidixic acid,[^18^, ^19^] enoxacin, flumequine, norfloxacin,[^20^, ^21^] ofloxacin,[^22^, ^23^] ciprofloxacin, pefloxacin, enrofloxacin, sparfloxacin, orbifloxacin, and cinoxacin, using triple quadrupole linear ion trap mass spectrometry.[^24^, ^25^, ^26^, ^27^] Speculative cleavage pathways are proposed, providing a reference for detecting and structuralizing quinolone antibiotics. 
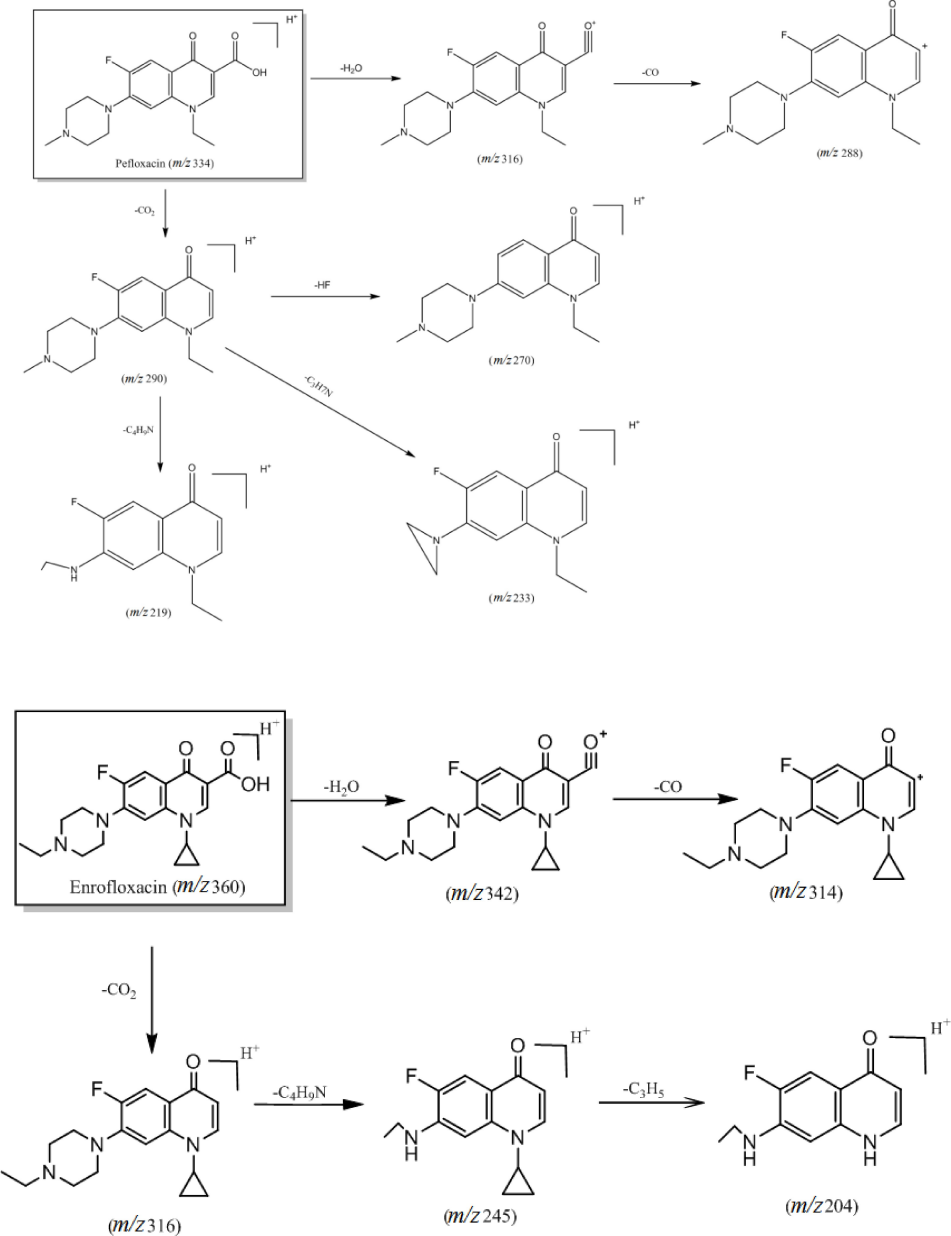
 
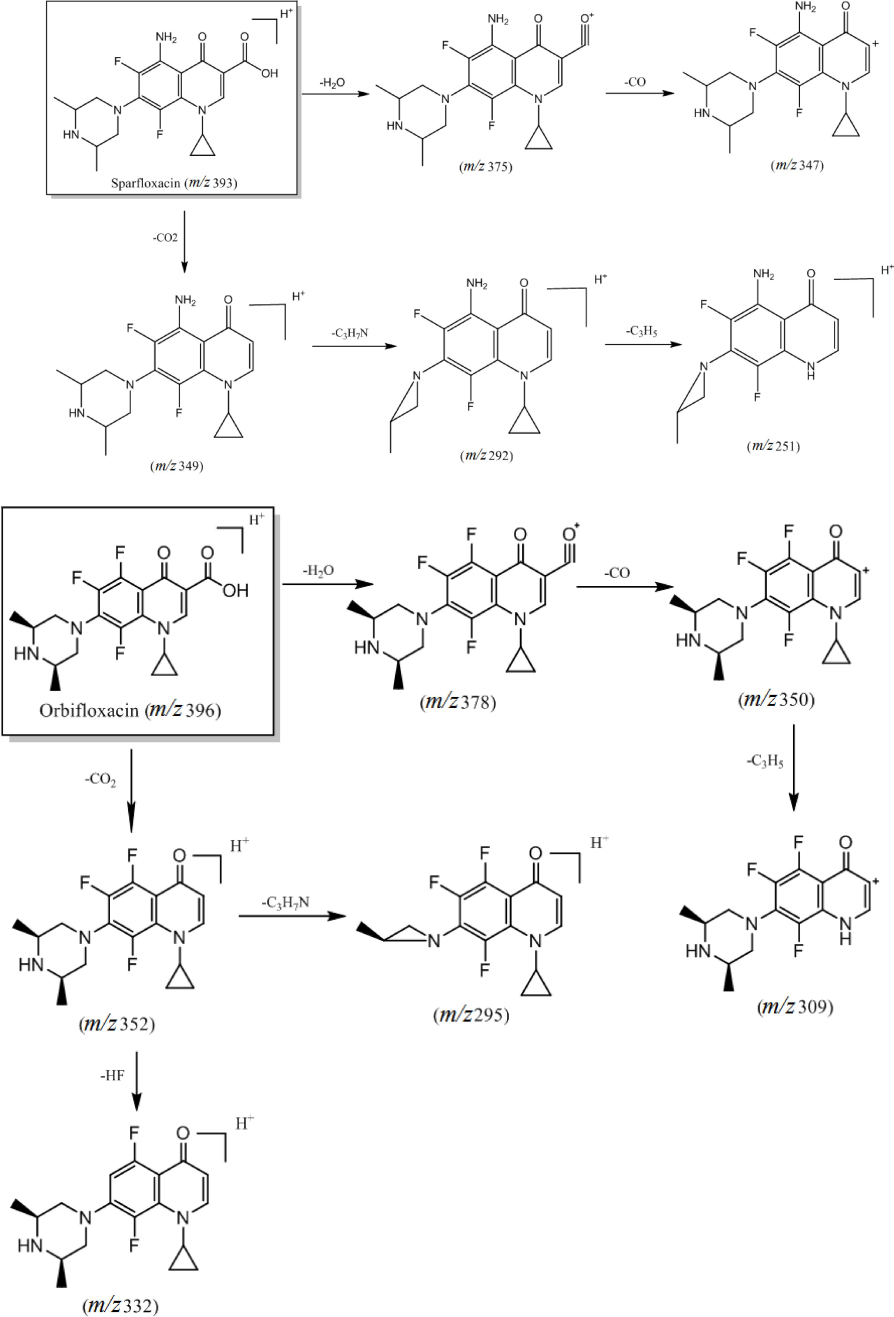


## Experimental

### Instruments and Reagents

Agilent 1290‐Sciex 5500QTRAP Triple Quadrupole Linear Ion Trap Mass Spectrometer (SCIEX, USA), equipped with an electrospray ionization source (ESI); Milli‐Q Ultra Pure Water Meter (Millipore, USA); Acetonitrile (Chromatographically Purity, Tedia, USA); Eleven quinolone standards (100 ug ⋅ mL^−1^, TMRM Quality Inspection, China): Nalidixic acid (CAS:389‐08‐2), Enoxacin (CAS:74011‐58‐8), Flumequine (CAS:42835‐25‐6), Ofloxacin (CAS:82419‐36‐1), Norfloxacin (CAS:70458‐96‐7), Ciprofloxacin (CAS:85721‐33‐1), Pefloxacin (CAS:70458‐92‐3), Enrofloxacin (CAS:93106‐60‐6), Sparfloxacin (CAS:111542‐93‐9), Orbifloxacin (CAS:113617‐63‐3), Cinoxacin (CAS:111542‐93‐9)

### Standard Solution Preparation

The mass concentration of the mixed standard reserve solution was 100 ng ⋅ mL^−1^, and 1 mL of each of the eleven quinolone standards (nalidixic acid, enoxacin, fluoromethane, ofloxacin, norfloxacin, ciprofloxacin, pefloxacin, enrofloxacin, sparfloxacin, ofloxacin, and cinoxacin) was measured precisely, and the solution was fixed with acetonitrile to 1000 mL, and then stored in a light‐proof environment at −18 °C for spare use.

### Instrument Parameters

Ion source: ESI electrospray ion source, positive ion mode was used for the scanning, Voltage: 5500 V, Curtain gas (CUR) pressure: 30 psi, Atomizing gas (GS1) pressure: 50 psi, Auxiliary gas (GS2) pressure: 60 psi, Temperature of the source (TEM): 500 °C, Collision gas (CAD): High. After confirming the parent ion through the first‐stage mass spectrometry (MS) scanning, we induced a collisional dissociation of quasi‐molecular ions [M+H]^+^. The collision energy CE was 25 eV±10 eV, and the fragment ions on the obtained secondary mass spectra were analyzed to study the cleavage characteristics and the cleavage pathway of the substance to be measured.

## 2 Structure and Discussion

All eleven quinolones feature N and C atoms in their parent nuclei, enabling them to accept protons readily; thus, all of them are well‐ionized in the positive ion mode, resulting in the formation of the quasi‐molecular ion peak of [M+H]^+^.

### 2.1 Similarities in Parent Nuclei Structure and Cleavage Pathways

**Figure 1:**
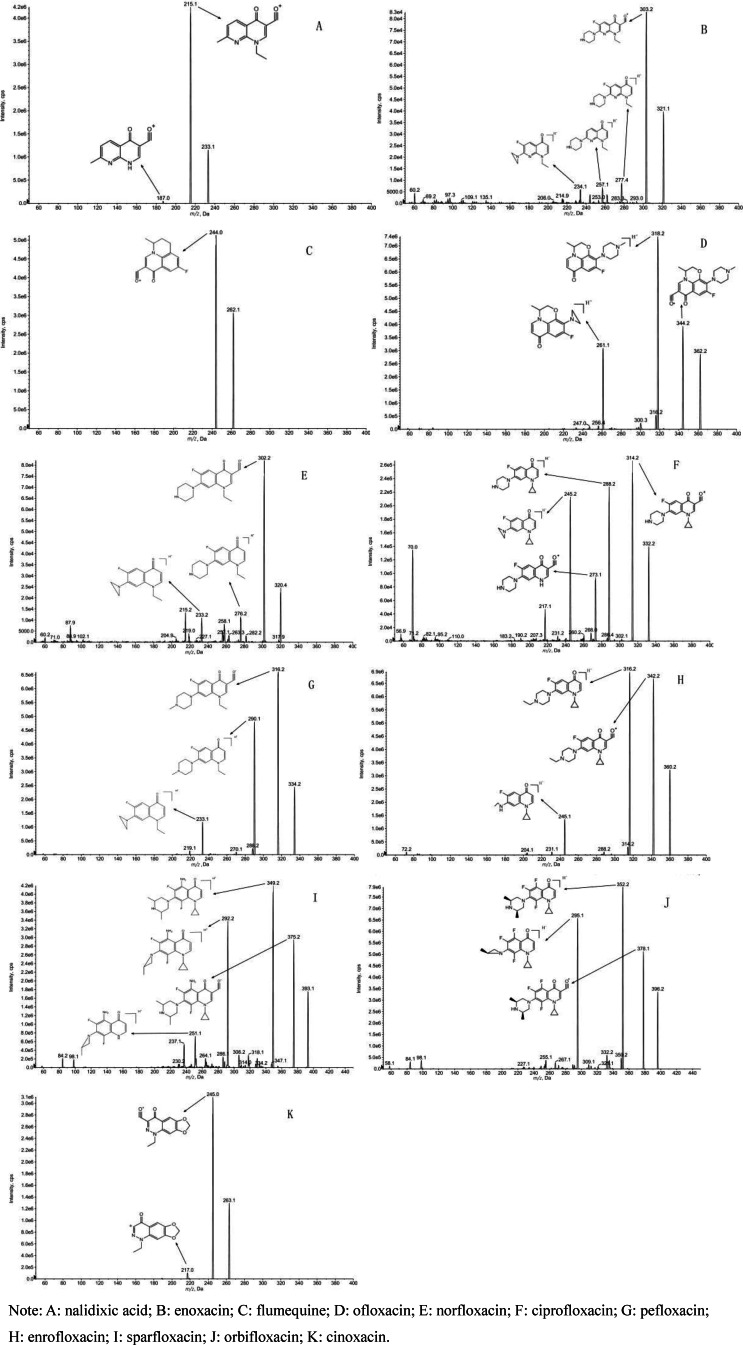
Selective ion flow mass spectra of mass standard solutions of 11 quinolone antibiotics.

The parent nuclei of quinolone antibiotics share common structural elements, including carboxyl groups at the C‐3 position and carbonyl groups at the C‐4 position. Mass spectral cleavage exhibits certain regularities due to these structural similarities. Notably, as all compounds in this class are substituted with carboxyl groups at the C‐3 position, a comparison of the structural and mass spectral information of these 11 compounds (refer to Figure 1) reveals characteristic ions such as [M+H−H_2_O]^+^, [M+H−CO]^+^, and [M+H−H_2_O−CO]^+^, which are readily observed in the soft ionization state. Detailed information on specific fragment ion peaks is presented in Table 1.

**Table 1:** Mass spectrometric cleavage fragmentation peaks of eleven quinolone antibiotics.

compound	nalidixic acid	enoxacin	flumequine	ofloxacin	norfloxacin	ciprofloxacin	pefloxacin	enrofloxacin	sparfloxacin	orbifloxacin	cinoxacin
[M+H]^+^	233	321	262	362	320	332	334	360	393	396	263
[M+H−H_2_O]^+^	215	303	244	344	302	314	316	342	375	378	245
[M+H−CO_2_]^+^		277		318	276	288	290	316	349	352	
[M+H−H_2_O−CO]^+^				316		286	288	314	347	350	217
[M+H−CO_2_‐HF]^+^		257				268	270			332	
[M+H−CO_2_‐C_2_H_5_N]^+^		234			233	245					
[M+H−H_2_O‐C_3_H_5_]^+^						273					
[M+H−H_2_O−CO‐C_3_H_5_]^+^										309	
[M+H−CO_2_‐C_3_H_7_N]^+^				261			233		292	295	
[M+H−CO_2_‐C_3_H_7_N‐C_3_H_5_]^+^									251		
[M+H−CO_2_‐C_4_H_9_N]^+^				247			219	245			
[M+H−CO_2_‐C_4_H_9_N‐C_3_H_5_]^+^								204			
[M+H−H_2_O‐C_2_H_4_]^+^	187										
[M+H−CO_2_‐C_2_H_5_N‐C_2_H_4_]^+^		206									
[M+H−H_2_O‐C_3_H_6_]^+^			202								

Note: A: nalidixic acid; B: enoxacin; C: flumequine; D: ofloxacin; E: norfloxacin; F: ciprofloxacin; G: pefloxacin; H: enrofloxacin; I: sparfloxacin; J: orbifloxacin; K: cinoxacin.

### 2.2 Differences in the Cleavage Patterns Due to Various Functional Groups

The eleven quinolone antibiotics were classified into four categories according to the functional groups attached to the parent nucleus, and the specific compound classification information is shown in Table 2. Mass spectrometry analysis was conducted to investigate the cleavage characteristics of these compounds and determine their cleavage patterns.

**Table 2:** Compound Classification Information.

First category	Compound Name	R_1_	R_5_	R_7_	R_8_
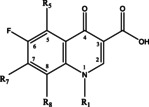	Norfloxacin	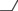	H	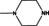	H
	Ciprofloxacin	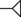	H	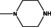	H
	Pefloxacin	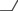	H	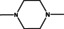	H
	Enrofloxacin	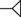	H	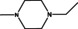	H
	Sparfloxacin	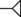	NH_2_	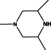	F
	Orbifloxacin	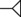	F	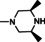	F

Second category	Compound Name	R_1_	R_6_	R_7_	
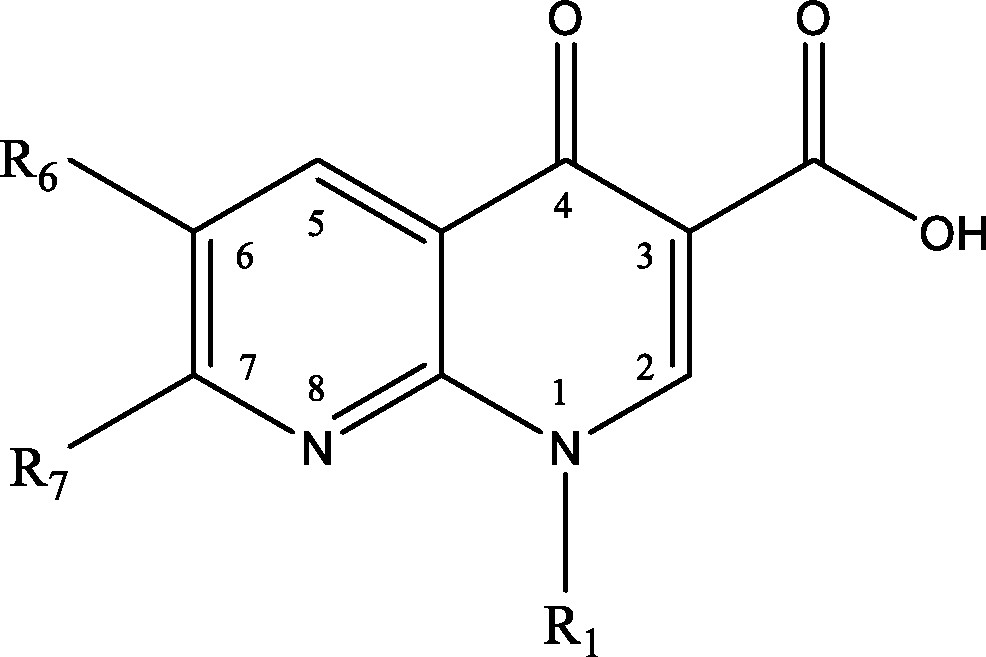	Nalidixic acid	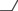	H	CH_3_	
	Enoxacin	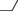	F	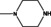	

Third category	Compound Name	X	R_7_		
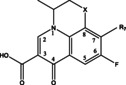	Flumequine	CH_2_	H		
	Ofloxacin	O	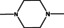		

Fourth category	Compound Name				
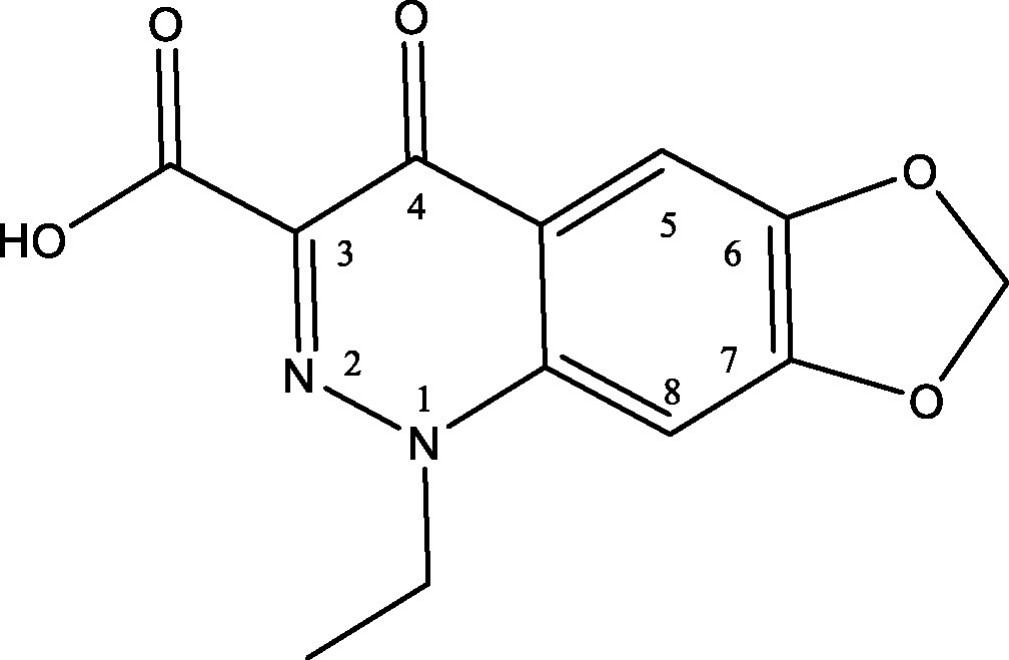	Cinoxacin				

The first category of quinolone antibiotics comprises norfloxacin, ciprofloxacin, pefloxacin, enrofloxacin, sparfloxacin, and orbifloxacin, all sharing similar structural features, including the introduction of F atoms at the C‐6 position, substitution of piperazine rings or piperazine derivatives at the C‐7 position, substitution of the C‐3 group with a carboxyl group, and substitution of the C‐4 group with a carbonyl group. The compounds in this group exhibit a consistent loss of 20 Da (HF) after dehydration or decarboxylation, in addition to characteristic fragment ion peaks such as [M+H−H_2_O]^+^, [M+H−CO]^+^ and [M+H−H_2_O−CO]^+^ resulting from dehydration or carboxyl group loss at the C‐3 position.

**Figure 2:**
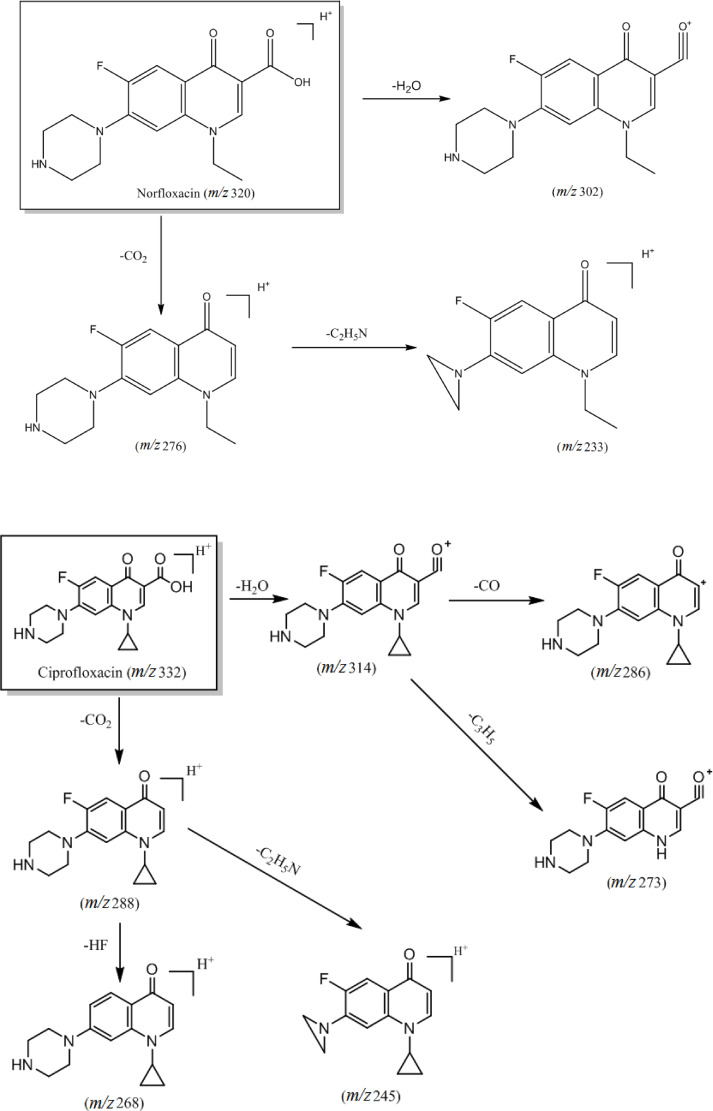
Cleavage pattern of quinolone antibiotics (category I).

The main differences within this class of compounds arise from the different types of substituents in the compound structure. For instance, substitutions at the C‐7 position with piperazine rings or derivatives result in the loss of neutral molecules such as 43 Da (C_2_H_5_N), 57 Da (C_3_H_7_N), and 71 Da (C_4_H_9_N) after fracture rearrangement yielding corresponding daughter ions. Moreover, compounds exhibit distinct cleavage patterns based on substituents at the N‐1 position, with cyclopropyl and ethyl substituents leading to observable differences in cleavage pathways. Notably, compounds with a cyclopropyl substituent tend to lose a cyclopropyl radical 41 Da (⋅C_3_H_5_) during cleavage to produce the corresponding daughter ion. The primary cleavage pathway is illustrated in Figure 2.

Analysis of the cleavage pathways of these six quinolone antibiotics reveals differing substituent groups at the N‐1 position. Specifically, ciprofloxacin, enrofloxacin, sparfloxacin, and orbifloxacin feature cyclopropyl substituents, while norfloxacin and pefloxacin have ethyl substituents. When a cyclopropyl group serves as a substituent, fracturing during the cleavage process leads to removing a cyclopropyl radical (⋅C_3_H_5_).^[13]^ The difference in the substituent groups at the C‐7 position lies in substituting ciprofloxacin and norfloxacin by a piperazine ring, which loses C_2_H_5_N after fracture rearrangement. Accordingly, enrofloxacin is substituted with an N‐ethylpiperazine ring, resulting in the loss of the C_4_H_9_N molecule after fracture rearrangement. Pefloxacin is substituted with an N‐methyl‐piperazine ring, while sparfloxacin and orbifloxacin are substituted with a dimethyl‐piperazine ring, all of which lose C_3_H_7_N after fracture rearrangement.

**Figure 3:**
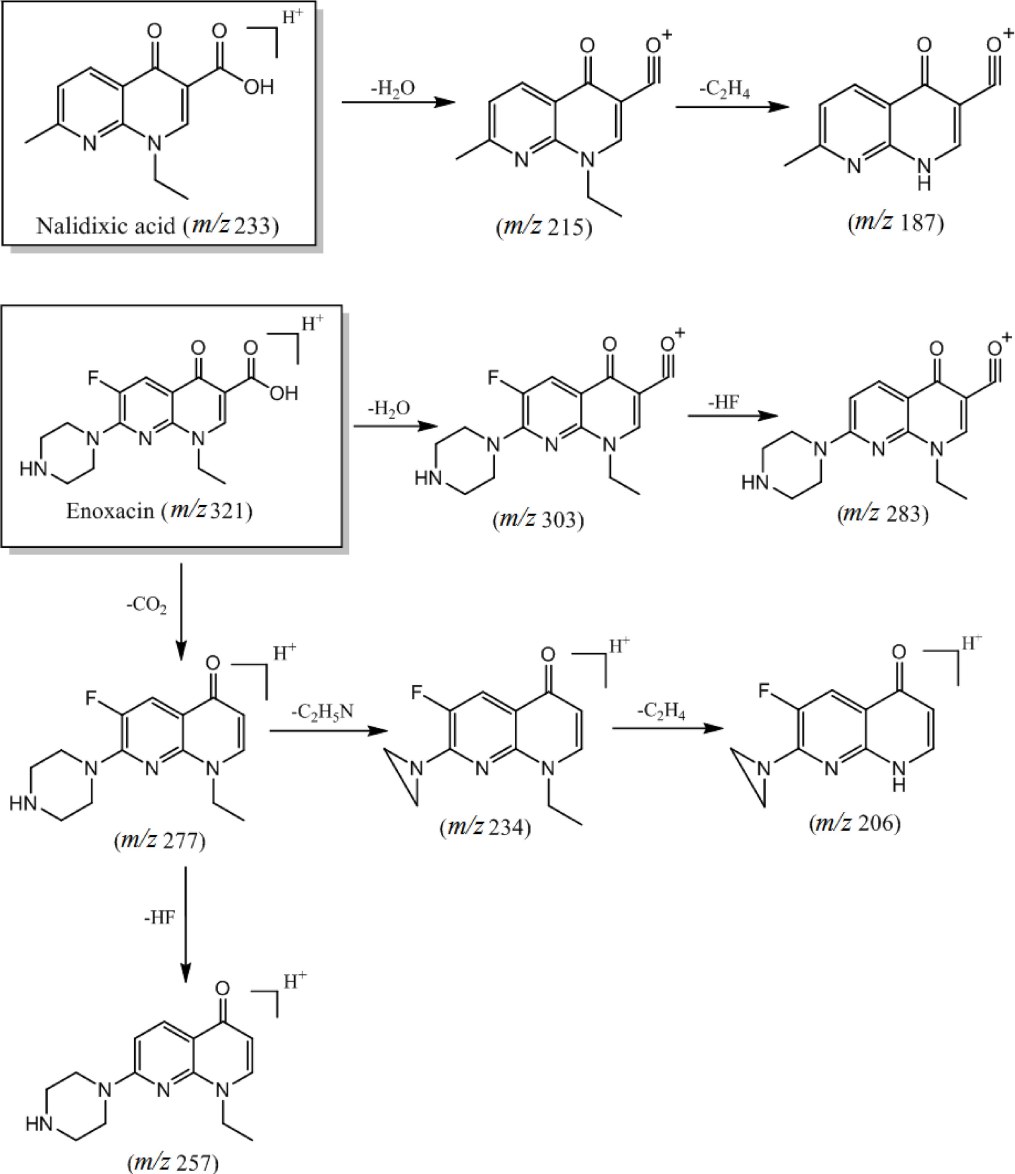
Cleavage pattern of quinolone antibiotics (category II).

The second category of quinolone antibiotics includes nalidixic acid and enoxacin. Their common structural characteristics feature the replacement of the 8‐position C atom of the parent nucleus by an N atom, the C‐3 substituent being a carboxyl group, and the N‐1 substituent being an ethyl group. Due to partial similarities in the structure of the parent nucleus, the [M+H−H_2_O]^+^ ion is produced by the dehydration of the carboxyl group at the C‐3 position, and the daughter ion was produced by the loss of the ethylene (C_2_H_4_) molecule due to breakage of the ethyl group at the N‐1 position. The main distinction between the two compounds is the substitution of enoxacin at the C‐6 position with F and the substitution of the C‐7 position with a piperazine ring. Analysis of the mass spectra of enoxacin (refer to Figure 1) suggests two distinct cleavage routes. One route involves the loss of an H_2_O molecule from the enoxacin quasi‐molecular ion peak (m/z 321) to form an ion (m/z 303), followed by the detachment of a molecule (HF) to produce a daughter ion (m/z 283). The other route involves the initial loss of a CO molecule from the excimer ion peak (m/z 321) to form the daughter ion (m/z 277), followed by the loss of a neutral molecule (HF 20 Da), or the breakage of a piperazine ring and its subsequent rearrangement to remove the neutral molecule (C_2_H_5_N 43 Da). The primary cleavage pathway is shown in Figure 3.

**Figure 4:**
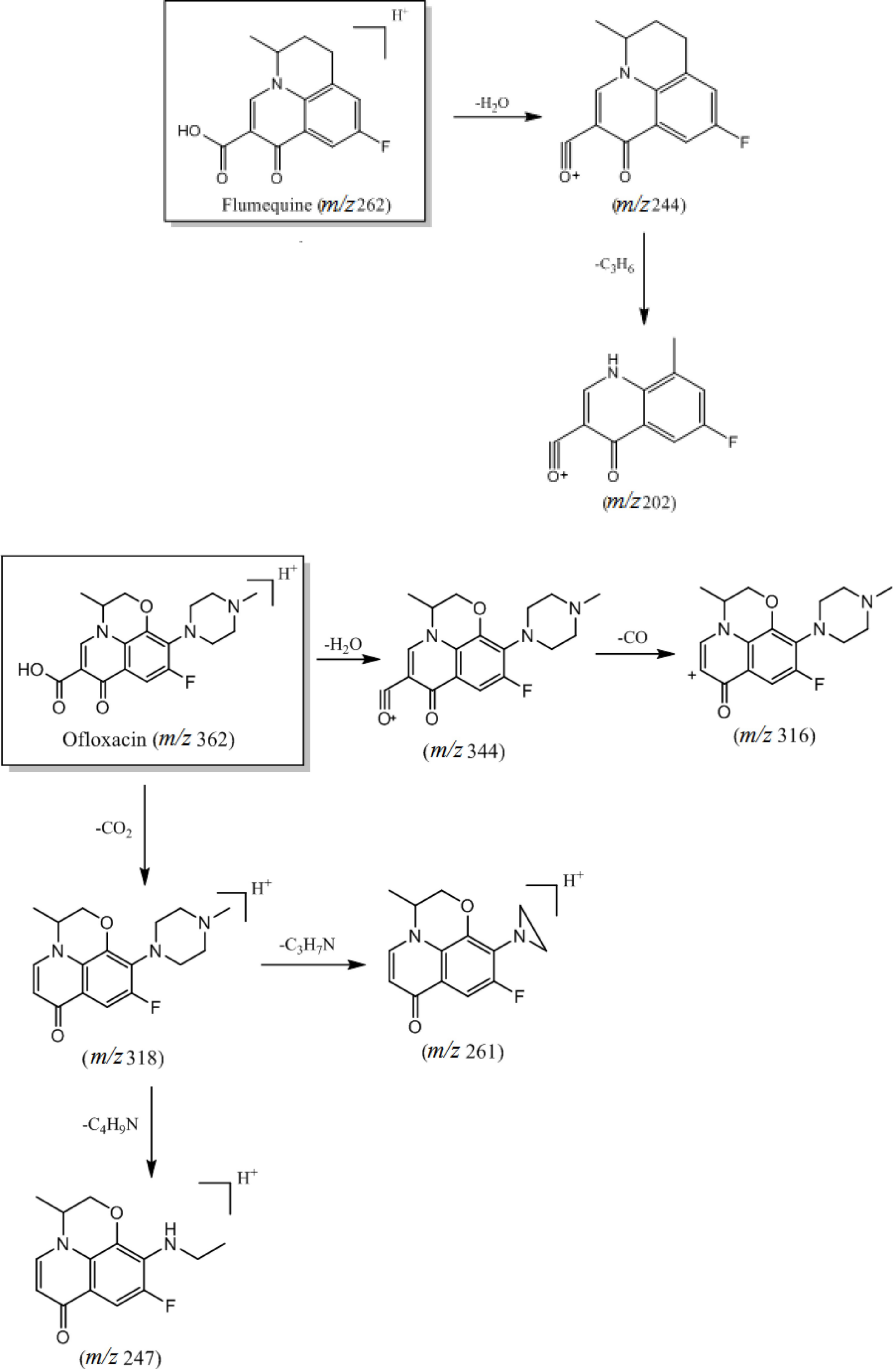
Cleavage pattern of quinolone antibiotics (category III).

The third category of quinolone antibiotics includes flumequine and ofloxacin, whose common structural features include introducing a carboxyl group at the C‐3 position of the parent nucleus and an F atom at the C‐6 position. These compounds exhibit the characteristic ion [M+H−H_2_O]^+^ in a soft ionized state; however, they vary in the cleavage patterns of their functional groups. In ofloxacin, the substituents at the N‐1 and C‐8 positions are interconnected to form a six‐membered ring, while the C‐7 position is substituted with an N‐methylpiperazine ring. This substitution involves partial rearrangement of the piperazine ring, leading to chain breakage and the removal of a neutral molecule (C_3_H_7_N or C_4_H_8_N), forming corresponding daughter ions. On the other hand, in fluoromethaquine, the O atom in the six‐membered ring is replaced by CH_2_ to form a six‐membered carbon ring. This reactive group can be readily cleaved to produce the neutral molecule C_3_H_6_, the loss of which generates a daughter ion at m/z 202. The primary cleavage pathways are illustrated in Figure 4.

**Figure 5:**
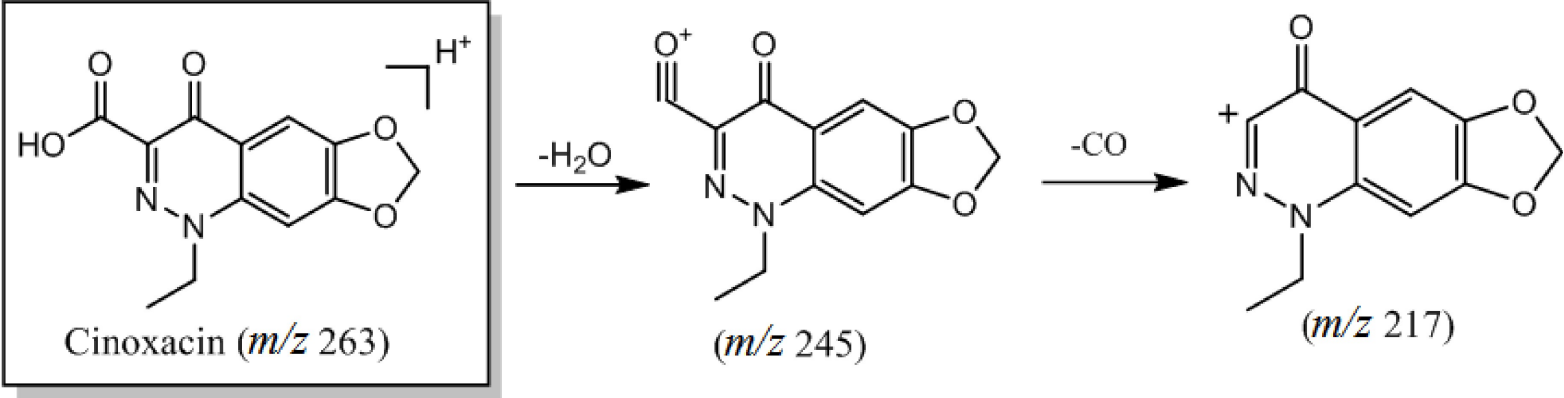
Cleavage pattern of quinolone antibiotics (category IV).

The fourth category of quinolone antibiotics is represented by cinoxacin, which exhibits significant structural differences compared to the top three categories. These differences primarily stem from introducing an F atom at the N‐2 position, while the C‐6 and C‐7 positions are linked by methylenedioxygen to form a ring. During the cleavage process, the quasi‐molecular ion peak (m/z 263) initially undergoes the removal of an H_2_O molecule, generating an ion at m/z 263. Subsequently, further cleavage leads to the loss of a neutral molecule (CO), yielding the daughter ion at m/z 217. The primary cleavage pathway is depicted in Figure 5.

### 2.3 Screening for Unknowns

**Figure 6:**
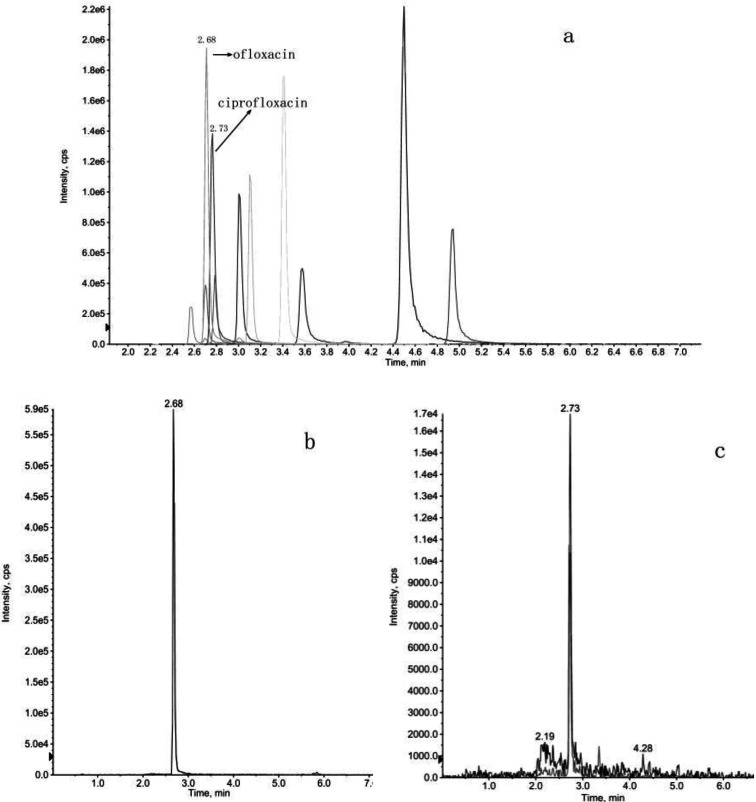
Total ion flow chromatograms of quinolone antibiotics in standard (a) and samples (b–c).

Usually, when detecting unknown samples, the total ion flow map of the target is easily masked by interferences and a large number of background ion signals, and there is great difficulty in identifying the signals by manual means.In order to obtain comprehensive information for the characterization of quinolone antibiotics in unknown samples, we used the IDA mode of LC–MS/MS. Chromatograms and mass spectral fragmentation ionograms in a single injection were collected simultaneously. At the same time, combine the cleavage pattern and fragment ions of quinolone antibiotics to identify the type of compounds quickly and comprehensively in the samples. Taking river sample as an example, samples were tested by MRM‐IDA‐EPI scanning mode.Then in combination with an established mass spectrometry spectral library to screen and analyse the samples. The chromatograms of the quinolone antibiotics in standards and samples are shown in Figure 6.

**Figure 7:**
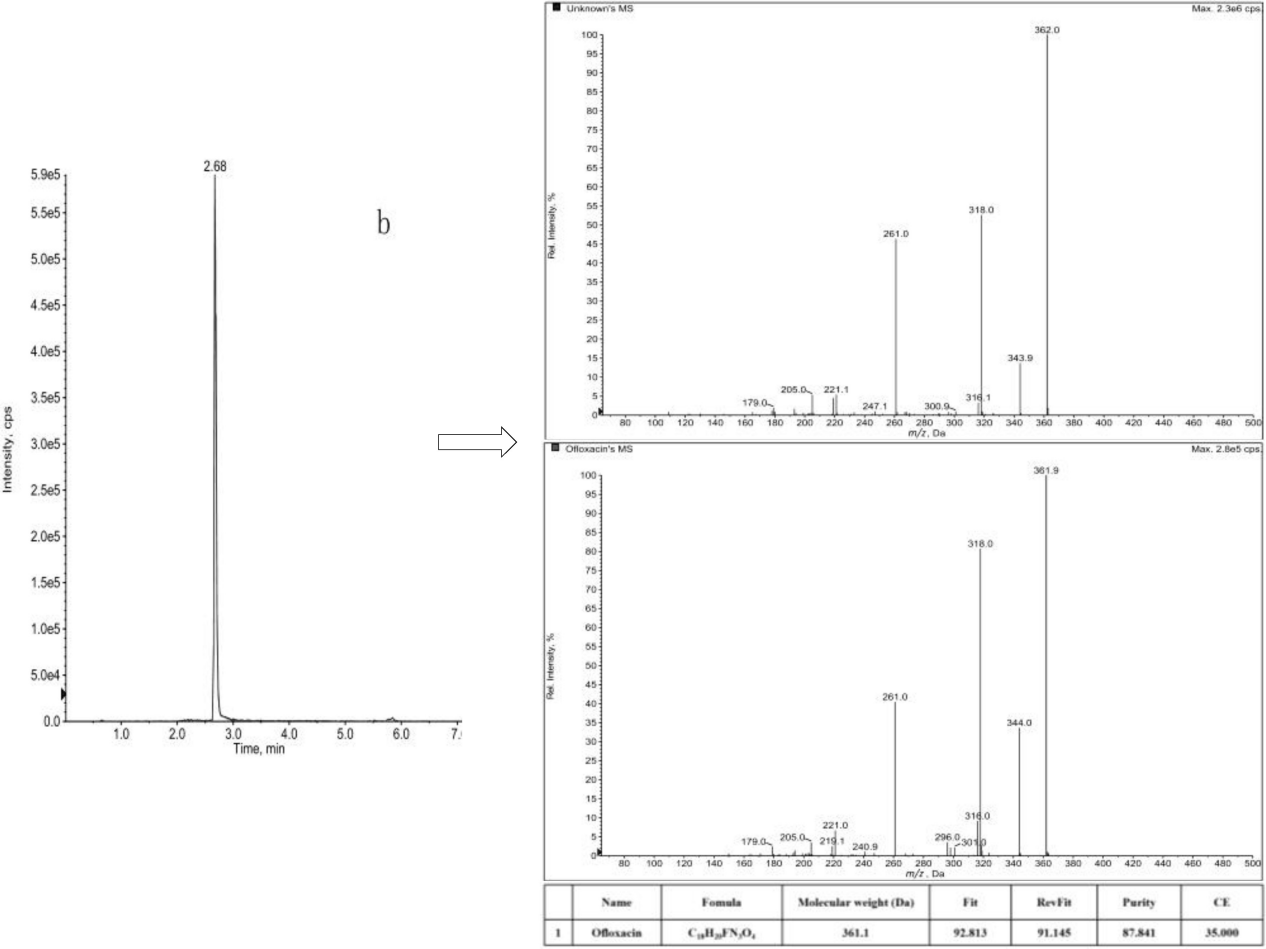
Mass spectrometric cleavage fragmentation of compound b in the sample.

Taking molecular ion (m/z 362) of the compound [M+H]^+^ as an example (Figure 7). The major fragment ions of the compound (m/z 362) in the sample are m/z 344 (removal of an H_2_O molecule), m/z 318 and m/z 261 (removal of CO_2_ followed by piperazine ring cleavage). It is consistent with the cleavage pattern and mass spectral fragmentation of ofloxacin standard. Combined with the time of the chromatographic peak and the result of mass spectrometry library, the Purity value was 87.841 %(Higher Purity values indicate greater confidence). It can be determined that Ofloxacin was detected in the sample.

**Figure 8:**
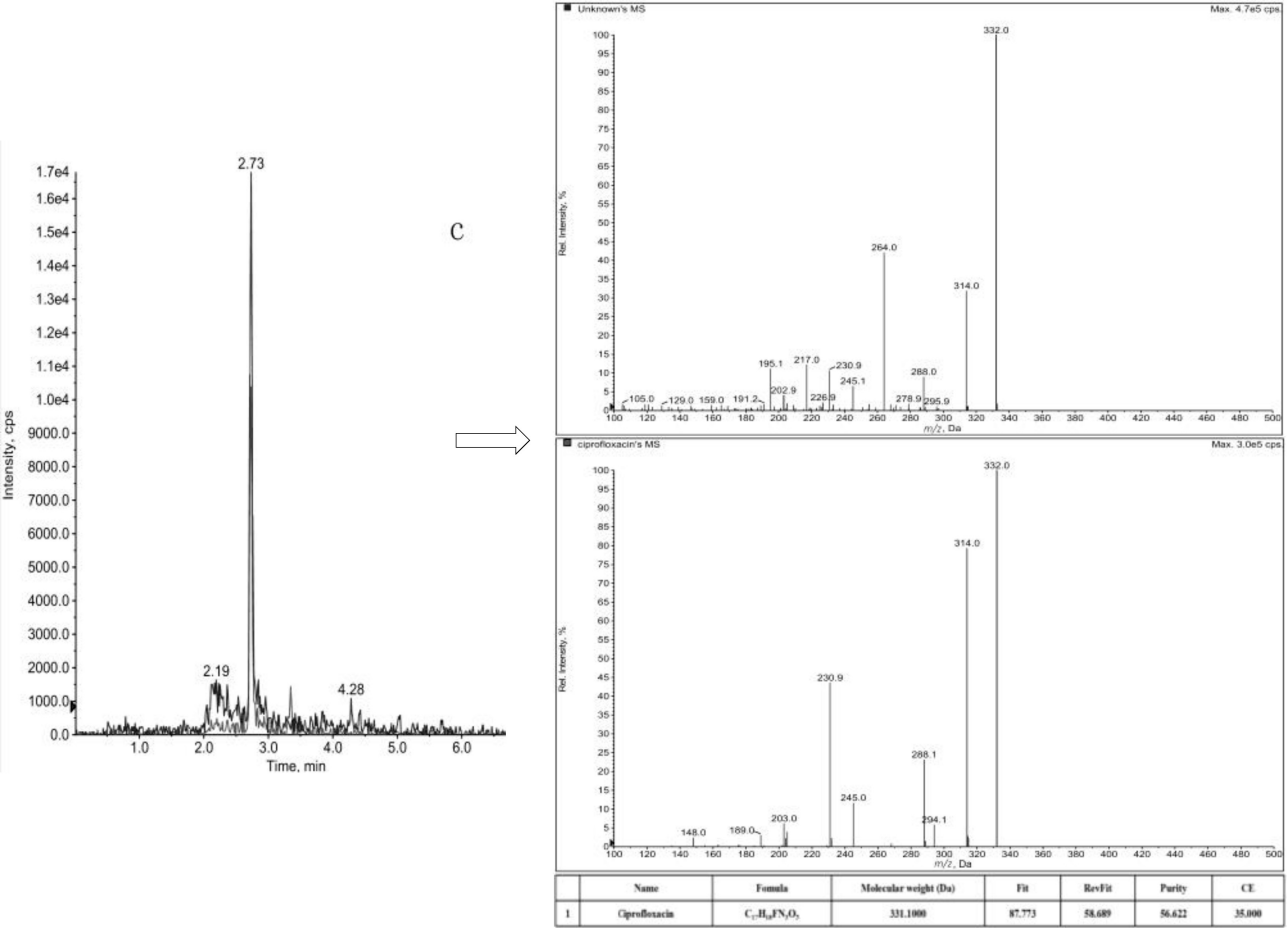
Mass spectrometric cleavage fragmentation of compound c in the sample.

Taking molecular ion (m/z 332) of the compound [M+H]^+^ as an example (Figure 8). The sample was found to have the same fragmentation ion m/z 314 (removal of one H_2_O molecule) as the standard ciprofloxacin, but lacked the other two major fragmentation ions, m/z 314 and m/z 245 (removal of CO_2_ followed by piperazine ring cleavage). Although the peak time of the compound was consistent with the ciprofloxacin, the possibility of ciprofloxacin was still excluded based on the fragment ions and 56.622 % of Purity value. The presence of false positives was effectively avoided by the cleavage pathway and cleavage fragments of mass spectrometry.

## 3 Conclusions

This study investigated the cleavage patterns of 11 quinolone antibiotics using triple‐quadrupole linear ion trap mass spectrometry. In the ESI+ mode, clear quasi‐molecular ion peaks of the protonated ion [M+H]^+^ were observed for all 11 compounds. Further collision‐induced cleavage with collision gas revealed characteristic fragmentation ions, including [M+H−H_2_O]^+^, [M+H−CO]^+^, and [M+H−H_2_O−CO]^+^, as well as fragmentation peaks indicative of the piperazinyl ring structure, which underwent deacidification followed by breakage and rearrangement to remove C_2_H_5_N, C_3_H_7_N, and C_4_H_8_N. Specifically, compounds featuring a cyclopropyl substituent at the N‐1 position exhibited characteristic fragmentation involving the continued loss of the cyclopropyl radical (⋅C_3_H_5_) following dehydration or deacidification. Additionally, when substituents at the N‐1 and C‐8 positions are interconnected to form a six‐membered carbocycle, the carbocycle could be easily broken to remove neutral CH molecules. In summary, although the 11 quinolone antibiotics shared similar parent nuclei and partially similar cleavage pathways, their distinctive functional groups induced varied cleavage patterns. These characteristic fragment ions provide an essential basis for the structural analysis of quinolone antibiotics with identical mother nucleus structures, facilitating the rapid screening and structural identification of novel quinolone antibiotics using mass spectrometry techniques.

## Conflict of Interests

The authors declare no conflict of interest.

4

## Data Availability

The data that support the findings of this study are available from the corresponding author upon reasonable request.
